# Stereotactic radiation therapy for liver metastases: factors affecting local control and survival

**DOI:** 10.1186/s13014-015-0369-9

**Published:** 2015-03-20

**Authors:** Nicolaus HJ Andratschke, Carsten Nieder, Franz Heppt, Michael Molls, Frank Zimmermann

**Affiliations:** Current Address: Department of Radiation Oncology, University Hospital Zurich, University of Zurich, Rämistrasse 100, 8006 Zurich, Switzerland; Department of Oncology and Palliative Medicine, Nordland Hospital, Bodø, Norway; Institute of Clinical Medicine, Faculty of Health Sciences, University of Tromsø, Tromsø, Norway; Institut für Radioonkologie, Medizinische Radiologie, Universitätsspital Basel, Basel, Switzerland; Department of Radiation Oncology, University of Rostock, Rostock, Germany; Department of Radiotherapy and Radiation Oncology, Klinikum rechts der Isar, Technische Universität München, Munich, Germany; Department of Dermatology, University Hospital Erlangen, Erlangen, Germany

**Keywords:** Stereotactic radiotherapy, SBRT, Liver, Metastases

## Abstract

**Purpose:**

To report on outcome and toxicity of stereotactic body radiotherapy (SBRT) for liver metastases in patients not eligible for surgery.

**Methods:**

From 2000 to 2009, 74 patients with 91 liver metastases from different primaries have been treated with SBRT at our institution. Median planning target volume was 123 ccm (range: 10.6-1074 ccm). Treatment consisted of 3–5 fractions with 5–12.5 Gy/ fraction prescribed to the surrounding 60-95% isodose with daily image guidance. Regular follow-up included CT or MRI imaging until tumor progression.

**Results:**

Median local recurrence-free interval was 23 months with a local control rate of 74.7%, 48.3% and 48.3% after 1, 2 and 3 years. Only minimum biologically effective dose (BED) to gross tumor volume (GTV) remained as independent significant factor for local control in multivariate analysis. No local recurrences were observed in lesions (n = 12) which received a minimal BED to the GTV of 120 Gy. Including 26 local recurrences, 67 patients (91%) showed disease progression after SBRT with a median time of 5 months. Median overall survival was 27 months with survival rates of 77%, 30% and 27% at 1, 3 and 5 years. On multivariate analysis only GTV volume remained as independent significant prognostic factor for overall survival (p = 0.002). No grade 3 to 5 acute toxicity and no grade 4 or 5 late toxicity occurred.

**Conclusion:**

SBRT for liver metastases was well tolerated in this non-selected patient cohort and yielded good local control despite the considerable size of most lesions treated. Long-term survival is possible after SBRT.

**Electronic supplementary material:**

The online version of this article (doi:10.1186/s13014-015-0369-9) contains supplementary material, which is available to authorized users.

## Introduction

Metastatic spread to the liver is quite a frequent event in the natural course of many common solid tumors [1-3]. Besides primary tumor site, histology, and extent of hepatic lesion(s), the presence of additional uncontrolled metastatic spread profoundly affects prognosis. In colorectal cancer, it has been shown that long term survival can be achieved with surgical treatment of solitary liver metastases as the only site of distant relapse [4,5]. Five-year survival rates in the order of 50% have been reported in highly selected patients [6]. Therefore, a treatment strategy focusing on effective local treatment may be indicated after proper patient selection. In more advanced, undoubtedly palliative cases, especially with other metastatic sites present - which may be stable on effective systemic treatment- local control of otherwise progressing liver metastases still may be necessary. In these patients with so-called oligo-progression, one tends to avoid aggressive surgical intervention. Thus, other effective approaches for local treatment need to be explored. The liver is one of the more radiosensitive organs with a low tolerance to large volume irradiation with regards to effective cytoablative radiation doses [7]. Therefore, radiotherapy has long been neglected as a locally effective and thus potentially curative or palliative alternative approach to resection or other invasive procedures like radiofrequency or laser ablation, cryosurgery or chemoembolisation.

Over the past 10 years, with rapid development of extracranial stereotactic radiation techniques and increasing knowledge of the radiobiology of the liver, especially tolerance to highly focused cytoablative radiation doses, stereotactic radiotherapy delivered either as single fraction or hypofractionated treatment has emerged as a promising alternative to surgical or interventional options in metastatic disease to the liver [8-13].

At the time of study initiation (year 2000), scarce clinical experience with large single fraction as used currently in SBRT protocols existed, with virtually no experience on the optimal prescription method (homogenous vs. inhomogenous dose distribution, level of inhomogeneity). Herein, we report on a single institutional experience in treatment of liver metastases by hypofractionated stereotactic radiotherapy (SBRT) in patients not eligible for surgical treatment. A normal-tissue adapted planning concept has been applied, using an inhomogenous dose prescription to the 60% isodose line covering the planning target volume, allowing a steep concentric dose built-up within the tumor.

## Patients and methods

### Patient eligibility

From December 2000 to September 2009, 76 patients with 95 metastatic lesions in the liver not eligible for surgical resection due to local tumor extension and/or patients comorbidities have been treated with SBRT at our institution. Indication for treatment included locally progressive liver disease which was not amenable to other local therapy, or progressive after chemotherapy and considered the predominant tumor burden endangering the patient in the short term. Therefore, besides patients with no evidence of extrahepatic disease, patients with stable or progressive extrahepatic disease were considered for liver SBRT as well. Patients with 1–4 liver metastases irrespective of histology were eligible after discussion in a multidisciplinary tumorboard, which had recommended against other treatment modalities including surgery.

Pretreatment investigations in all patients consisted of physical examination, laboratory tests including blood counts and liver enzymes, computed tomography (CT) scan of the thorax and abdomen with i.v. contrast and - in selected patients - whole body positron emission tomography (PET, Tracer: 18-F-fluorodeoxyglucose (FDG)) on a dedicated combined PET/CT hybrid scanner.

### Radiation treatment planning and delivery

Immobilization for image acquisition and treatment was carried out in a vacuum couch with a low-pressure foil (Medical Intelligence GmbH, Schwabmünchen, Germany). An abdominal compression device was used which aimed at reducing the motion of the diaphragm. During CT scanning and irradiation patients received oxygen supply to further reduce respiratory motion.

Gross tumor volume (GTV) was contoured as the visible tumor in the planning CT supplemented by information from magnetic resonance imaging (MRI). No separate CTV margin was applied (i.e. GTV = CTV). Breathing motion was taken into account by sequential CT scans to derive an individual margin for each patient. This internal target volume was complemented by an additional margin of 5 mm (axial dimension) and 10 mm (longitudinal dimension) to account for reproducibility of patient positioning.

After January 2009, patients (n = 15) additionally received a 4D-CT and a respiration correlated PET scan in treatment position. All imaging modalities (planning CT, MRI, 4D-PET-CT) were then fused using iPlanNet V4 (Brainlab AG, Feldkirchen, Germany) and a composite GTV contour was generated using the image information on macroscopic tumor from all image modalities. PTV margins consisted of the tumor motion in all dimensions as derived from the 4D-PET-CT complemented by an isotropic margin of 5 mm in all directions to account for setup uncertainties.

Treatment consisted of 3–5 fractions with 5–12.5 Gy per fraction prescribed to the surrounding 60-95% isodose (median 35 Gy in 5 fractions to the 60% isodose) using 5–8 coplanar open static fields. The most common regimens were 35 Gy in 5 fractions (44 lesions; Dmax = 58.3 Gy, BED = 126 Gy_10_), 30 Gy in 3 fractions (10 lesions; Dmax = 50 Gy, BED = 133 Gy_10_), 37.5 Gy in 3 fractions (8 lesions; Dmax = 62.5 Gy, BED = 193 Gy_10_) and 30 Gy in 5 fractions (5 lesions; Dmax = 50 Gy), all prescribed to the 60% isodose line. The following considerations led us to prescribe to a lower isodose line compared to other groups. At the time this study was initiated, scarce evidence for any fractionation and prescription mode was available (year 2000). Therefore we hypothesized that at the PTV margin an EQD2Gy of 50–70 Gy, depending on the fractionation schedule applied, should be sufficient to control microscopic disease and a steep concentric dose increase should allow for significantly higher doses within the tumor. At the same time, a more rapid dose fall off outside the target may facilitate adherence to normal tissue constraints. Whenever feasible with regards to normal tissue constraints, the 60% isodose line had to fully cover the PTV. Otherwise, minimum dose to the PTV and corresponding prescription isodose line were chosen depending on the given dose constraint for the gastrointestinal tract and liver: Dmax (bowel, stomach): 5×5.4 Gy or 3×7.0 Gy, Dmax (esophagus): 5×6Gy or 3×9 Gy, D33% (liver) < 5×3.5 Gy or <3×5 Gy.

Positioning of the immobilized patient and target localization was verified by additional CT scans with subsequent transport of the patient within the immobilization couch to the linac (before July 2008), or by Cone Beam CT (after July 2008) before every treatment fraction.

### Follow-up

During treatment, all patients were monitored daily for acute treatment related toxicity. Follow-up 6 weeks after completion of SBRT and every 3–4 months thereafter included blood count, serum liver parameters and CT and/ or MRI scans until tumor progression.

Acute toxicity was scored according to the National Cancer Institute CTCAE v3.0 criteria during and up to 3 months after radiotherapy. Late toxicity was graded using the RTOG/EORTC criteria.

Local failure of a metastatic lesion was defined as either reappearance after complete remission or re-growth after initial partial response in follow-up CT or MRI scans.

Extrahepatic tumor status was classified as either no evidence of disease (NED), stable (SD) or progressive disease (PD).

### Statistical analysis

Actuarial survival time and time to other endpoints were calculated according to the Kaplan-Meier method. For univariate and multivariate analysis of prognostic factors Cox proportional hazard model was used. For overall survival any death and for disease specific survival death from the underlying cancer was defined as an event. For actuarial local tumor control, progression of the treated lesion was defined as stated in the Methods section. For this endpoint, patients who died from other diseases without tumor regrowth or progression at that time were censored. All time intervals were calculated from the last day of SBRT. Biological effective dose was calculated according to the LQ formalism: BED = n * d * (1 + d/ {α/β}) with n being the number of fractions, d the daily single fraction dose and alpha-beta for tumor tissue of 10 Gy.

Comparison of survival between groups was performed using the log-rank test. Statistical analysis was performed with PASW Statistics V18 (SPSS Inc./IBM, Somer, NY) and the R statistical environment version 2.12.2.

## Results

This analysis included 38 male and 36 female patients with a median age of 61 years (range 40–80 years). Two patients with 2 liver metastases each had to be excluded as they were lost to follow up immediately after radiotherapy. Of the 95 lesions, 91 were evaluable for local control and 89 for the influence of tumor volume (GTV) and dose on local control. Table 1 summarizes the patient and treatment characteristics. The median Karnofsky performance status (KPS) was 90 (range 50–100). 37 patients had colorectal cancer (50%), 12 patients breast cancer (16%), and 25 patients other primary tumors (34%). Thirty-five patients (47%) either had extrahepatic stable or progressive disease, the remainder showed no evidence of disease outside the liver. SBRT was the initial treatment of liver metastases in 23 of 91 lesions (25%). Forty-four metastases (48%) were previously exposed to chemotherapy and 3 (3%) to endocrine treatment. Resection, other local ablative procedures or combinations of these had been performed in the remaining cases. Of these 68 pre-treated lesions 47 showed clinical progression before SBRT, while 21 were irradiated for residual lesions as identified on CT or MRI. The median GTV size was 45 cc (range 1.3-699 cc). The median PTV size was 123 cc (range 11–1074 cc). In 10 patients (13.5%) more than 1 target volume was irradiated. All patients completed their planned course of SBRT. Median follow-up was 15 months. Forty-two patients (56.7%) had died at the time of this analysis.

**Table 1:** **Patient and treatment characteristics**

		**Patients**
No. of patients evaluable/completing treatment		74/76
Gender	Male	38
	Female	36
Age at treatment (years)	Median	61 (range 40-80)
ECOG performance status	Median	0 (range 0-2)
0		50
1		22
2		2
Status of extrahepatic disease		
NED (no evidence of disease)		39 (53%)
SD/PD (stable/progressive)		35 (47%)
Previous treatment per irradiated lesion		
No previous treatment		23 (25.3%)
Chemotherapy		44 (48.4%)
Endocrine therapy		3 (3.3%)
Local therapy (surgery, radiofrequency ablation)		4 (4.4%)
Combination of local and systemic treatment		17 (18.6%)
Follow up (months)	Median	15 (range 3-103)
Primary tumor	Colorectal	37
	Breast	12
	Esophageal	5
	Stomach/Pancreas/Bile duct	7
	Lung	2
	Other	11
		**Lesions**
Total No. of lesions evaluated/treated		91/95
Lesions per patient	Mean	1.23 (range 1-4)
Total PTV dose at 60-95% IDL	Median	35 (range 18-37.5)
(Gy)		
Single PTV dose at 60-95% IDL	Median	7 (range 5-12.5)
(Gy)		
Fractions	Median	5 (range 2-5)
Minimum BED to GTV	Median	91.2 Gy (18.7-183.5Gy)
Gross tumor volume GTV (cc)	Median	45 (range 1.3-699)
<100		65
100-200		12
>200		12
Planning target volume PTV (cc)	Median	123 (range 10.6-1074)

### Local control

**Figure 1:**
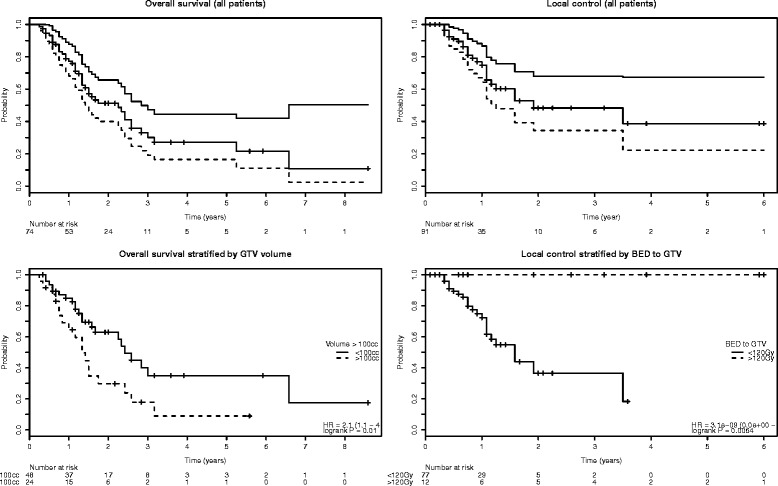
**Kaplan-Meier estimates for overall survival (upper left panel), stratified by GTV volume (lower left panel), local control (upper right panel), stratified by BED to the GTV (lower right panel).** Bounding curves in the upper panel represent the 95% confidence interval.

Median local recurrence free interval for all patients was 23 months with a local control rate of 74.7%, 48.3% and 48.3% after 1, 2 and 3 years, respectively (Figure 1). Only minimal biologically effective dose (either to GTV {p < 0.0001} or PTV {p = 0.01}) and tumor volume (p = 0.03) were predictive for local control in univariate analysis (Table 2). In multivariate analysis only the minimal BED to the GTV remained as independent prognostic factor for local control of the irradiated lesions (p = 0.015). No local recurrences were observed in lesions (n = 12) which received a minimal BED to the GTV of 120 Gy.

**Table 2:** **Univariate and multivariate analysis for local control and overall survival according to patient and tumor characteristics (** ^**§**^ primary treatment vs progression after pretreatment; &complete remission/stable disease vs progressive disease; *colorectal cancer vs. other; **volume and minimum biologically effective dose as continuous variables; ECOG performance status 0 vs. 1-2; abbreviations: GTV=gross tumor volume, PTV=planning target volume, PS= performance status, HR=hazard ratio, CI=95% confidence interval)

**Local control**				
	**Univariate**		**Multivariate**	
	**HR (CI)**	**p-value**	**HR (CI)**	**p-value**
**Indication for SBRT** ^**§**^	0.91 (0.42-1.99)	0.82	0.97 (0.35-2.72)	0.96
**Prior CTx**	0.66 (0.25-1.79)	0.42	0.33 (0.08-1.30)	0.11
Histology*	1.69 (0.72-3.98)	0.22	1.58 (0.61-4.10)	0.35
GTV Volume**	1.003 (1-1.005)	0.03	1.002 (0.99-1.004)	0.27
BED PTV**	0.95 (0.92-0.99)	0.01	0.98 (0.93-1.03)	0.38
BED GTV**	0.97 (0.96-0.99)	<0.001	0.98 (0.96-0.996)	0.015
**Overall survival**				
	**Univariate**		**Multivariate**	
	**HR (CI)**	**p-value**	**HR (CI)**	**p-value**
**ECOG PS**	0.71 (0.38-1.34)	0.29	0.92 (0.53-2.25)	0.83
Histology*	0.53 (0.29-0.98)	0.04	0.65 (0.32-1.34)	0.25
**Prior CTx**	0.58 (0.26-1.33)	0.19	0.70 (0.26-1.87)	0.47
**Extrahepatic disease status before SBRT&**	2.5 (1.3-4.8)	0.005	1.69 (0.81-3.51)	0.16
GTV Volume**	1.003 (1.001-1.004)	<0.001	1.003 (1.001-1.004)	0.002
**Local recurrence after SBRT**	0.80 (0.42-1.52)	0.5	0.70 (0.34-1.48)	0.35

### Pattern of failure

Of 44 patients with tumor progression in the liver, 26 had local recurrences of SBRT treated metastases. Of the 44 patients, 24 showed intrahepatic failure only. Another 23 patients developed extrahepatic progression only. Overall, 67 patients (91%) showed disease progression after SBRT after a median time of 5 months (Table 3).

**Table 3:** **Patterns of failure and treatment of progression after index treatment**

	**Total (n=74)**
**Dead**	42
**Any disease progression**	67
**Local progression at treated site**	26
**Location of first progression**	
Liver only	24
Liver and other organs	20
Other organs only	23
**Treatment for first progression**	
Local Treatment	17
Chemotherapy	43
Others	7

### Survival

Median overall survival was 27 months with survival rates of 77%, 30% and 27% at 1, 3 and 5 years (Figure 1). Eleven patients survived more than 36 months after SBRT. In univariate survival analysis extrahepatic disease status (NED/SD vs. PD; p = 0.005), colorectal primary (p = 0.04) and smaller GTV volume (p < 0.001) were correlated significantly with better overall survival (Table 2). On multivariate analysis only GTV volume remained as independent significant prognostic factor for overall survival (p = 0.002).

### Toxicity

Treatment was very well tolerated with only grade 1–2 acute side effects including predominantly fatigue (20.3%), nausea and vomiting (17.7%), fever (6.8%), skin reaction (5.4%) and upper abdominal pain (8.1%). Rare events included grade II pneumonitis (n = 1; antibiotic and steroid therapy) and cholecystitis (n = 1; antibiotic therapy). No grade 3 to 5 acute toxicity occurred (Table 4).

**Table 4:** **Acute and late side effects according to CTCAE v3.0**

**Acute toxicity**	**Grade 1-2**
GI tract	17 (23%)
Nausea	10 (13.6%)
Vomiting	3 (4.1%)
Inappetence	1 (1.4%)
Diarrhea	0
Obstipation	2 (2.7%)
Meteorism	1 (1.4%)
Skin reaction	4 (5.4%)
Fatigue	15 (20.3%)
Fever	5 (6.8%)
Abdominal pain	6 (8.1%)
Pneumonitis	1 (1.4%)
Cholecystitis	1 (1.4%)
**Late toxicity**	**Grade 1-2**
GI tract	10
Nausea	4
Vomiting	1
Inappetence	3
Diarrhea	1
Meteorism	1
Skin reaction	5*
Fatigue	8
Abdominal pain	5
Pneumonitis	3
Rib fracture	5

(*One patient experienced skin reaction with a local ulcer which required local excision).

Relevant long-term complications included pneumonitis grade 2 (n = 2), skin ulcer (n = 1; grade 2, local surgery required; thoracic wall infiltration by the metastases) and rib fractures (n = 5; only analgetics necessary). No grade 4 or 5 late toxicity occurred.

## Discussion

So far, surgical resection is still considered the gold standard for operable liver metastases in functionally operable patients, if a significant benefit with regard to quality of life and overall survival is to be expected [14,15]. Still, not all patients presenting with a limited number of liver metastases in an oligometastatic setting are suitable candidates for resection. Over the past two decades several local ablative therapies have emerged, with radiofrequency ablation being the most widely used [16]. In recent years, SBRT - originally developed for ablative high-precision radiotherapy of brain tumors – has been adopted for treatment of primary or secondary tumors of the lung and liver. Our institution started liver SBRT in December 2000. Patients were referred by one of the hospital’s multidisciplinary tumor boards, typically either the gastrointestinal or breast cancer board. Most patients were pre-treated for their liver metastases and had quite large lesions but were in good general condition (Table 1). Here we report on long-term follow-up of all patients treated during the first 9 years.

Our retrospective analysis suggests that both long-term local control and survival is possible, although a significant proportion of patients relapsed outside and inside of the irradiated region. Acceptable toxicity rates and pattern were seen, despite treatment of multiple and/or large lesions in many patients (no radiation-induced liver disease). The latter was also the case in the phase II trial recently reported by Scorsetti et al. [17]. They had treated 61 patients with 76 lesions (3 fractions) and observed excellent local control and survival at 1 year (both >80%). In a variety of other publications, results were largely comparable, as summarized in Additional file 1: Table S1 [8-13,17-21]. In the phase I portion of a trial reported by Rusthoven et al., dose was escalated from 36 Gy to 60 Gy in three fractions, in increments of 6 Gy, without dose-limiting toxicity [11]. In the phase II component, the dose was 60 Gy in three fractions prescribed to the 80-90% isodose line. Thirteen patients were treated to doses less than 60 Gy, and 36 patients received 60 Gy. For lesions with maximum diameter of 3 cm or less, 2-year local control was 100% compared with 77% for lesions greater than 3 cm (p = 0.015). Berber et al. have recently evaluated a multicenter database from 4 academic medical centers in the United States (April 2000 - September 2010, different techniques, total dose 37.5 ± 8.2 Gy in 5 ± 3 fractions, 70% isodose line) [22]. They reported on 153 patients (363 tumors, most primaries were of colorectal or breast origin). The mean GTV size was 138 cc, range 8–581 cc). Mean follow-up was 25 months. One-year survival was 51% (77% in our study) and local control was 62% (75% in our study). Grade 3 and 4 toxicity was observed in 3% of patients (no deaths from SBRT). These results support our own and various previous studies, demonstrating that SBRT is a safe technique for the precise delivery of radiation to liver tumors.

Chang et al. reported on a pooled patient cohort treated with SBRT for colorectal liver metastases (1–4 lesions, 1–6 fractions of SBRT) [23]. Their series included 65 patients with 102 lesions from 3 institutions. The median follow-up was 1.2 years. Total dose, dose per fraction and BED all correlated with local control. The estimated dose range needed for 1-year local control >90% was 46–52 Gy in 3 fractions. We have also seen favorable local control in all metastases treated to a high BED (BED of greater than 120 Gy with an alpha/beta of 10 Gy). However, dependent on size, number of targets and dose-limiting normal tissues such high BED cannot always be achieved. Under these circumstances, SBRT to lower doses might still provide valuable palliation, preventing for example biliary obstruction. Given that most patients progress somewhere in the liver or in other organs within 12 months, it is clear that local measures cannot be considered curative in the majority of patients. Predictive factors are needed to identify those patients who are unlikely to relapse at other sites and for whom optimal local control is a prerequisite for long-term survival, comparable to selection criteria for surgical candidates. Most likely, these factors vary with primary disease type or even subgroup as in the case of colorectal (isolated metastases in the liver) and breast cancer (triple negative, Her2 positive etc.), making it necessary to perform much larger studies than hitherto. It is also important to define the dose–response relationships and optimal fractionation regimens in a more concise and definitive manner. Randomized trials comparing different fractionation schedules and even well-designed prospective phase-II-trials are still lacking. Given our toxicity data, it appears safe to use the dose constraints that we applied, i.e. Dmax (bowel, stomach): 5x5.4 Gy or 3x7.0 Gy, Dmax (esophagus): 5x6Gy or 3x9 Gy, D33% (liver) < 5x3.5 Gy or <3x5 Gy. However, as local and locoregional control rates need to be further improved, alternatives might also be considered, for example the dose constraint that 700 cc of normal liver would receive less than 15 Gy, to allow a further dose escalation to the metastatic lesions. Rusthoven et al. recommended a maximum total dose to any point in the spinal cord and stomach/small intestine not to exceed 18 Gy and 30 Gy, respectively (3 fractions) [11]. Importantly, one should recognize that true long-term toxicity is not well defined because published series have short median follow-up and few long-term survivors.

SBRT proved as an effective local treatment option for technically and medically inoperable patients with up to 4 liver metastases without major side effects observed so far. Even when large tumors or metastases in very critical locations within the liver have to be treated by SBRT, and very strict dose constraints are used for radiosensitive structures and organs, a respectable local control rate can be achieved. Side effects have not been critical and treatment was very well tolerated. Local control was mainly influenced by lesion size and radiation dose, while overall survival was predominantly affected by lesion size and extrahepatic disease control. Unfortunately, systemic progression still limits long-term survival in this poor prognostic group.

## Conclusion

Based on these initial results after implementation of liver SBRT, it seems reasonable to treat up to 4 metastases regardless of tumor volume in ablative intent as long as normal tissue constraints are respected, as even patients with large tumor volumes can achieve substantial survival after SBRT. Nevertheless, if the tumor volume (based on GTV) as the single most important factor predicting overall survival exceeds 100 cc (approx. 6 cm diameter) or a sufficient GTV dose cannot be achieved (<120Gy BED), realistic treatment aims have to be communicated to the patient (temporary response, limited prolongation of survival).

## Additional file

Additional file 1: Table S1. Comparison with published international results [8-13,17-21].

## References

[1] LeGolvan MP, Resnick M. Pathobiology of colorectal cancer hepatic metastases with an emphasis on prognostic factors. J. Surg. Oncol. Wiley Subscription Services, Inc., A Wiley Company; 2010;102:898–908. 10.1002/jso.21817 21165991

[2] Kakeji Y, Morita M, Maehara Y (2010). Strategies for treating liver metastasis from gastric cancer. Surg Today Springer Japan;.

[3] Pagani O, Senkus E, Wood W, Colleoni M, Cufer T, Kyriakides S (2010). International guidelines for management of metastatic breast cancer: can metastatic breast cancer be cured?. J Natl Cancer Inst.

[4] Ciferri E, Bondanza GS, Municinò O, Castagnola M, Gazzaniga GM (2003). Colorectal cancer metastases: surgical indications and multimodal approach. Hepatogastroenterology.

[5] Foster JH (1990). Surgical treatment of metastatic liver tumors. Hepatogastroenterol.

[6] Isoniemi H, Osterlund P (2011). Surgery combined with oncological treatments in liver metastases from colorectal cancer. Scand J Surg.

[7] Pan CC, Kavanagh BD, Dawson LA, Li XA, Das SK, Miften M (2010). Radiation-associated liver injury. Int J Radiat Oncol Biol Phys.

[8] Vautravers-Dewas C, Dewas S, Bonodeau F, Adenis A, Lacornerie T, Penel N (2011). Image-guided robotic stereotactic body radiation therapy for liver metastases: is there a dose response relationship?. Int J Radiat Oncol Biol Phys.

[9] Kavanagh BD, Schefter TE, Cardenes HR, Stieber VW, Raben D, Timmerman RD (2006). Interim analysis of a prospective phase I/II trial of SBRT for liver metastases. Acta Oncol.

[10] Herfarth KK, Debus J, Lohr F, Bahner ML, Rhein B, Fritz P (2001). Stereotactic single-dose radiation therapy of liver tumors: results of a phase I/II trial. J Clin Oncol.

[11] Rusthoven KE, Kavanagh BD, Cardenes H, Stieber VW, Burri SH, Feigenberg SJ (2009). Multi-Institutional Phase I/II Trial of Stereotactic Body Radiation Therapy for Liver Metastases. J Clin Oncol.

[12] Lee MT, Kim JJ, Dinniwell R, Brierley J, Lockwood G, Wong R (2009). Phase I Study of Individualized Stereotactic Body Radiotherapy of Liver Metastases. J Clin Oncol.

[13] Wulf J, Guckenberger M, Haedinger U, Oppitz U, Mueller G, Baier K (2006). Stereotactic radiotherapy of primary liver cancer and hepatic metastases. Acta Oncol.

[14] Johnston FM, Mavros MN, Herman JM, Pawlik TM (2013). Local therapies for hepatic metastases. J Natl Compr Canc Netw.

[15] Alberts SR (2012). Update on the optimal management of patients with colorectal liver metastases Critical Reviews in Oncology/Hematology. Elsevier.

[16] Mahnken AH, Pereira PL, de Baère T (2013). Interventional oncologic approaches to liver metastases. Radiology.

[17] Scorsetti M, Arcangeli S, Tozzi A, Comito T, Alongi F, Navarria P (2013). Is stereotactic body radiation therapy an attractive option for unresectable liver metastases? A preliminary report from a phase 2 trial. Int J Radiat Oncol Biol Phys.

[18] Rule W, Timmerman R, Tong L, Abdulrahman R, Meyer J, Boike T (2011). Phase I dose-escalation study of stereotactic body radiotherapy in patients with hepatic metastases. Ann Surg Oncol.

[19] van der Pool AEM, Méndez Romero A, Wunderink W, Heijmen BJ, Levendag PC, Verhoef C (2010). Stereotactic body radiation therapy for colorectal liver metastases. Br J Surg.

[20] Katz AW, Carey-Sampson M, Muhs AG, Milano MT, Schell MC, Okunieff P (2007). Hypofractionated stereotactic body radiation therapy (SBRT) for limited hepatic metastases. Int J Radiat Oncol Biol Phys.

[21] Méndez Romero A, Wunderink W, Hussain SM, De Pooter JA, Heijmen BJ, Nowak PC, Nuyttens JJ, Brandwijk RP, Verhoef C, Ijzermans JN, Levendag PC (2006). Stereotactic body radiation therapy for primary and metastatic liver tumors: A single institution phase i-ii study. Acta Oncol.

[22] Berber B, Ibarra R, Snyder L, Yao M, Fabien J, Milano MT (2013). Multicentre results of stereotactic body radiotherapy for secondary liver tumours. HPB (Oxford).

[23] Chang DT, Swaminath A, Kozak M, Weintraub J, Koong AC, Kim J (2011). Stereotactic body radiotherapy for colorectal liver metastases: a pooled analysis. Cancer.

