# Supplementary material for: Stereotactic radiation therapy for liver metastases: factors affecting local control and survival
Source: Radiat Oncol. 2015 Mar 20;10:69. doi: 10.1186/s13014-015-0369-9 (PMC4464868; doi:10.1186/s13014-015-0369-9)
Supplement: Additional file 1: Table S1. — Comparison with published international results [8-13,17-21]. [file 13014_2015_369_MOESM1_ESM.doc]

Additional file 1: Table S1: Comparison with published international results

|  |  | |  | |  |  | | |  |  |  |  | | | |  | | |  | |  |  |  |  | | |  |  |  |
| --- | --- | --- | --- | --- | --- | --- | --- | --- | --- | --- | --- | --- | --- | --- | --- | --- | --- | --- | --- | --- | --- | --- | --- | --- | --- | --- | --- | --- | --- |
|  | Author | | Year | | No. of patients (No. of metastases) | Median tumor volume (GTV;cm³) | | | SRS or HFSRT (No. of fractions) | Dose (Gy) | Median Follow-up (months) | Local Control | | | Overall survival | | | | |  | | | | | | | | | |
|  |  |  |  |  |  |  |  |  |  |  |  |  |  |  |  |  |  |  |  |  |  |  |  |  |  |  |  |  |  |
|  |  |  |  |  |  |  |  |  |  |  |  | Median (months) | 1 year (%) | 2 years (%) | Median (months) | | 1 year (%) | 2 years (%) | |  | | | | | | | | | |
|  | Scorsetti et al. [17] | | 2013 | | 61 (76) | 18.6¶ | | | HFSRT (3) | 52.5-75 | 12 | - | 94 | 91 | 19 | | 83.5 | 18  months: 65 | |  | | | | | | | | | |
|  | Vautravers-Dewas et al. [8] | | 2011 | | 42 (62) | 36 | | | HFSRT (3-4) | 40-45 * | 14.3 | - | 90 | 86 | - | | 94 | 48 | |  | | | | | | | | | |
|  | Rule et al. [18] | | 2010 | | 27 (36) | 8.2 † | | | HFSRT (3-5) | 30-60 * | 20 | - | 56-100 | 56-100 | 37 | | - | 50-67 | |  | | | | | | | | | |
|  | Van der Pool et al. ‡ [19] | | 2010 | | 20 (31) | 6.4 † | | | HFSRT (3) | 37.5-45 | 26 | 22 | 100 | 74 | 34 | | 100 | 83 | |  | | | | | | | | | |
|  | Lee et al. [12] | | 2009 | | 68 | 75.2 | | | HFSRT (6) | 27.7-60 * | 10.8 | - | 71 | - | 17.6 | | 60 | 18 months: 47 | |  | | | | | | | | | |
|  | Rusthoven et al. [11] | | 2009 | | 47 (63) | 10.3 † | | | HFSRT (3) | 36-60 * | 16 | - | 95 | 92 | 20.5 | | - | 30 | |  | | | | | | | | | |
|  | Katz et al. [20] | | 2007 | | 69 (174) | 10.3 † | | | HFSRT (7-20) | 30-55 | 14.5 | - | 10 months: 76 | 20 months: 57 | 14.5 | | 10 months: 78 | 20 months: 37 | |  | | | | | | | | | |
|  | Kavanagh et al. [9] | | 2006 | | 21 (28) | 14.3 | | | HFSRT (3) | 60 | 19 | - | 18 months: 93 | | - | | - | - | |  | | | | | | | | | |
|  | Mendez-Romero et al. [21] | | 2006 | | 17 (34) | - | | | HFSRT (3) | 30-37.5 | 12.9 | - | 100 | 86 | - | | 85 | 62 | |  | | | | | | | | | |
|  | Wulf et al. ¦[13] | | 2006 | | 39 (51) | 53¶ | | | HFSRT (3-4) / SRS (1) | 26-37.5 * | 15 | - | 92 | 66 | 16 | | 72 | 32 | |  | | | | | | | | | |
|  | Herfarth et al. [10] | | 2005 | | 43 (66) | 18 | | | SRS (1) | 14-26 Gy | 6.6 | - | 18 months: 82 | | 24 | | 76 | 59 | |  | | | | | | | | | |
|  | **Own Data** | | **2013** | | **74 (91)** | **45** | | | **HFSRT (2-5)** | **18-40 (Median 35)** | **15** | **23** | **75** | **48** | **27** | | **77** | **51** | |  | | | | | | | | | |
|  |  | |  | |  |  | | |  |  |  |  |  |  |  | |  |  | |  | | | |  |  |  | | | |
|  | * Dose escalation | |  | |  |  | | |  |  |  |  |  |  |  | |  |  | |  | | | |  |  |  | | | |
|  | † Tumor volume (cm³) was recalculated by formula V = 1/6 x π x diameter³. | | | | | | | |  |  |  |  |  |  |  | |  |  | |  | | | |  |  |  | | | |
|  | ¥ Tumor diameter was recalculated. | | | | | |  |  | |  |  |  |  |  |  | |  |  | |  | | | |  |  |  | | | |
|  | ¦ Only data of patients with secondary liver tumors are listed. | | | | | |  |  | |  |  |  |  |  |  | |  |  | |  | | | |  |  |  | | | |
|  | ¶ Only median CTV is shown. | | |  | | |  |  | |  |  |  |  |  |  | |  |  | |  | | | |  |  |  | | | |
|  | | Median tumor volume was recalculated. | | | | | |  |  | |  |  |  |  |  |  | |  |  | |  | | | |  |  |  | | | |
|  | £ Overall survival was recalculated. | | |  | | |  |  | |  |  |  |  |  |  | |  |  | |  | | | |  |  |  | | | |
|  | GTV: Gross Tumor Volume |  | |  | | |  |  | |  |  |  |  |  |  | |  |  | |  | | | |  |  |  | | | |
|  | SRS: Stereotactic Radiosurgery | | |  | | |  |  | |  |  |  |  |  |  | |  |  | |  | | | |  |  |  | | | |
|  | HFSRT: Hypofractionated stereotactic radiotherapy | | | | | |  |  | |  |  |  |  |  |  | |  |  | |  | | | |  |  |  | | | |
